# Efficacy and safety of neoadjuvant systemic therapy in resectable hepatocellular carcinoma: a Systematic Review and meta-analysis

**DOI:** 10.3389/fonc.2025.1504917

**Published:** 2025-05-09

**Authors:** Dongdong Wu, Ning Liu, Hao Dong, Kan Zhou, Lei Du, Ying Li, Yanjun Chao, Fuping Ma

**Affiliations:** Department of Hepatobiliary Surgery, Xianyang Central Hospital, Xianyang, Shaanxi, China

**Keywords:** hepatocellular carcinoma, neoadjuvant, systemic therapy, resectable, systematic review, meta-analysis

## Abstract

**Background:**

Neoadjuvant systemic therapy has been shown to benefit patients with solid tumors such as breast cancer and colorectal cancer, but its application in hepatocellular carcinoma (HCC) is still in the exploratory stage, with no established effective regimen. This systematic review and meta-analysis aims to investigate the efficacy and safety of neoadjuvant systemic therapy in patients with resectable HCC.

**Methods:**

The clinical trials of resectable HCC neoadjuvant systemic therapy in PubMed, Embase and the Cochrane Library were systematically searched. A meta-analysis was performed using STATA/MP18.0 software, and the effect size was calculated using either a fixed effects model or a random effects model, and 95% confidence intervals (CIs) were calculated. Subgroup analysis was performed according to the neoadjuvant systemic therapy regimen.

**Results:**

This meta-analysis included 328 patients from 15 studies. In patients with resectable HCC, the pooled pathologic complete response (pCR) rate was 15% (95%CI: 10%–21%), the major pathologic response (MPR) rate was 28% (95%CI: 21%–35%), the incidence of grade 3–4 treatment-related adverse events (TRAEs) was 11% (95% CI: 4%–20%), the objective response rate (ORR) was 27% (95% CI: 20%–35%), the surgical resection rate was 84% (95%CI: 75%–92%), and the delay rate was 0.00% (95% CI: 0%–4%). The results of subgroup analysis showed that the efficacy of targeted therapy combined with immunotherapy is superior to dual ICI (immune checkpoint inhibitor) combination therapy and ICI monotherapy, while the safety of the ICI monotherapy was the highest, superior to the dual ICIs and the targeted therapy combined with immunotherapy.

**Conclusion:**

Neoadjuvant systemic therapy shows preliminarily beneficial outcomes in resectable HCC treatment. However, future large-scale and multicenter randomized controlled trials are needed to confirm this conclusion.

**Systematic review registration:**

https://www.crd.york.ac.uk/prospero/, identifier CRD42024562257

## 1 Introduction

Primary liver cancer is one of the most common malignant tumors worldwide, and it is also the main cause of cancer-related death, which seriously threatens human life and health (1, 2). Hepatocellular carcinoma (HCC) is the most common pathological type of primary liver cancer, accounting for about 75%-90% (3, 4). At present, radical surgical resection is still the first choice for HCC treatment, but the postoperative recurrence rate is high, and the 5-year recurrence rate is as high as about 70%; the survival time is short, and the 5-year survival rate of early stage HCC after operation is only 60% (5). Therefore, prolonging the survival period of patients and reducing the risk of postoperative recurrence are urgent problems to be solved.

Neoadjuvant therapy is an effective treatment strategy for patients with resectable malignant tumors with initial surgical opportunities to reduce tumor volume, reduce distant metastasis, increase R0 resection rate and reduce postoperative recurrence, so as to improve the survival of patients (6, 7). At present, it has been proved to be effective in a variety of solid tumors (8–10). Neoadjuvant therapy for HCC mainly includes local therapy represented by vascular intervention and systemic therapy represented by targeted therapy and immunotherapy (11). The role of neoadjuvant therapy in the treatment of HCC is still unclear, and it is still in the exploratory stage. The current guidelines have not clearly recommended any kind of neoadjuvant therapy for HCC (12, 13).

This meta-analysis tries to evaluate the efficacy and safety of neoadjuvant systemic therapy in resectable HCC by collecting existing clinical research data, aiming to provide some reference for preoperative neoadjuvant therapy in clinical patients with HCC.

## 2 Materials and methods

This systematic review and meta-analysis was conducted according to the Preferred Reporting Items for Systematic Reviews and Meta-Analyses (PRISMA) statement (14). The protocol of the systematic review has been prospectively registered on PROSPERO (CRD42024562257).

### 2.1 Search strategy and eligibility criteria

A comprehensive literature search was conducted for studies published from the inception of databases to 1 Jun 2024, in PubMed, Embase and the Cochrane Library to identify relevant studies. Search subject terms included (“ hepatocellular carcinoma “OR” HCC “OR” liver cancer “OR” primary liver carcinoma “) AND (“ liver resection “OR “surgical resection” OR “hepatectomy” OR “resectable”) AND (“ neoadjuvant “OR” perioperative “[Title] OR “preoperative” [Title]) and related entries (as detailed in the **Supplementary Material**). The references of the search results were further searched to prevent missing detection.

The inclusion criteria are as follows: (1) clinical studies consist of resectable adult HCC patients (aged > 18 years); (2) at least one form of targeted therapy and immunotherapy should be used as neoadjuvant systemic therapy before surgery; (3) studies to evaluate the efficacy and safety of neoadjuvant systemic therapy include at least one of the following main outcome indicators: pathological complete response (pCR), major pathological response (MPR), grade 3–4 treatment-related adverse event rate (Grade 3–4 TRAEs), objective response rate (ORR), surgical resection rate, and surgical delay rate; (4) studies by the same author only include recent publications or studies with large sample sizes and high quality.

Articles would be excluded if (1) they are duplicate publications; (2) they represent animal experiments, reviews, case reports or meta-analyses; (3) effective outcome indicators could not be obtained in the study(such as pCR, MPR, Grade 3–4 TRAEs, ORR, surgical resection rate, and surgical delay rate); (4) the study included non–primary HCC patients or patients with other malignant neoplastic diseases; (5) patients had received previous systemic therapy (e.g. targeted therapy, immunotherapy).

### 2.2 Data collection and quality assessment

Two reviewers independently(NL and HD) conducted the identification and extraction of potentially eligible articles. Any discrepancies were resolved by involving a third reviewer(KZ). Subsequently, the identified articles were retrieved, and a comprehensive analysis of their full texts was performed. For each study, a range of data was meticulously recorded, encompassing details such as the first author, publication year, trial number, country, sample size, study stage, intervention, study type, article type, treatment protocol, primary outcome measures (including pCR, MPR, incidence of grade 3–4 TRAEs, ORR, surgical resection rate, surgical delay rate).In cases where this data was unavailable, calculations were made based on the information provided within the articles. Data on these outcomes were collected from each eligible study, and any instances of missing data were noted. When data were incomplete, our team made efforts to contact the corresponding author for clarification. Data were extracted from the included studies using a standardized template developed by the reviewers and maintained in Microsoft Excel (Microsoft, Redmond, WA, USA). Inter-observer agreement was assessed using Cohen’s kappa (κ=0.85).

Since all but one of the included studies were single-arm studies with no control group, the quality of the studies was assessed according to the Methodological Index for Non–randomized Studies, (MINORS) (15) scale. Two reviewers conducted the assessment independently and resolved any differences through discussion with the third reviewer. The MINORS scale contains 12 items, of which items 9–12 are used to evaluate studies with control groups. Scores range from 0–2 for each item, 0 indicated unreported, 1 reported but inadequately, and 2 reported and fully detailed. The quality assessment of the included studies is shown in **Table 1**.

**Table 1:** Quality assessment of included studies.

Study	I	II	III	IV	V	VI	VII	VIII	IX	X	XI	XII	SCORE
Shi, Y.H. et al., 2021 (16)	2	2	2	2	2	2	2	0	–	–	–	–	14
Ho, W.J. et al., 2021 (17)	2	2	2	2	2	2	2	0	–	–	–	–	14
Chen, S. et al., 2022 (18)	2	2	2	2	2	2	2	0	–	–	–	–	14
D’Alessio, A. et al., 2022 (19)	2	2	2	2	2	2	2	0	–	–	–	–	14
Marron, T.U. et al., 2022 (20)	2	2	2	2	2	2	2	0	–	–	–	–	14
Kaseb, A.O. et al., 2022 (21)	2	2	2	2	2	2	2	0	2	2	2	2	22
Xia, Y. et al., 2022 (22)	2	2	2	2	2	2	2	0	–	–	–	–	14
Bai, X. et al., 2022 (23)	2	2	2	2	2	2	2	0	–	–	–	–	14
Su, Y. et al., 2023 (24)	2	2	2	2	2	2	2	0	–	–	–	–	14
Sun, H.C. et al., 2023 (25)	2	2	2	2	2	2	2	0	–	–	–	–	14
Song, T.Q., 2023 (26)	2	2	2	2	2	2	2	0	–	–	–	–	14
Cheung, T. T. et al., 2023 (27)	2	2	2	2	2	2	2	0	–	–	–	–	14
Huang, Z. et al., 2023 (28)	2	2	2	2	2	2	2	0	–	–	–	–	14
T. Song., 2024 (29)	2	2	2	2	2	2	2	0	–	–	–	–	14
Ming, K. et al., 2024 (30)	2	2	2	2	2	2	2	0	–	–	–	–	14

I, Clearly stated aim; II, Inclusion of consecutive patients; III, Prospective collection of data; IV, Endpoints appropriate to the aim of the study; V, Unbiased assessment of the study endpoint; VI, Follow-up period appropriate to the aim of the study; VII, Loss to follow up less than 5%; VIII, Prospective calculation of the study size; IX, Appropriateness of Control Group Selection; X, Synchrony of Control Group; XI, Comparability of Baseline Characteristics; XII, Adequacy of Statistical Methods. The items are scored 0 (not reported), 1 (reported but inadequate) or 2 (reported and adequate).

In the table, only the study by Kaseb, A.O.et al., is a randomized controlled trial,necessitating assessments for appropriateness of control group selection, synchrony of control group, comparability of baseline characteristics, and adequacy of statistical methods. The remaining fourteen studies are single-arm trials or case series studies, hence they do not require the aforementioned four assessments, and are marked with a hyphen (–).

### 2.3 Statistical analysis

STATA/MP18.0 software (StataCorp LLC, College Station, TX, USA) was used for statistical analysis of all data. Chi-square test and *I^2^* statistic were used for heterogeneity. If there was significant heterogeneity (*P*<0.1 and *I^2^* >50%), a random-effects model was used; otherwise, a fixed-effects model was used. We also performed subgroup analyses to explore the source of heterogeneity and whether there were differences between treatment regiments. Statistical comparison between subgroups was performed using the Cochran’s Q test to assess heterogeneity between groups. For analyses that included more than two subgroups, we determined the significance of the between-group difference by calculating the Q statistic and its corresponding *P* value. The percent weight was calculated using the inverse variance method, where the weight of each study was the inverse of the variance of its effect size, and the sum of all weights was standardized to 100%. In addition, to assess potential publication bias, funnel plots were used, and Egger’s test was used to evaluate whether the funnel plots were symmetric. *P*<0.1 indicated statistically significant differences.

## 3 Results

### 3.1 Literature search results and basic characteristics

**Figure 1:**
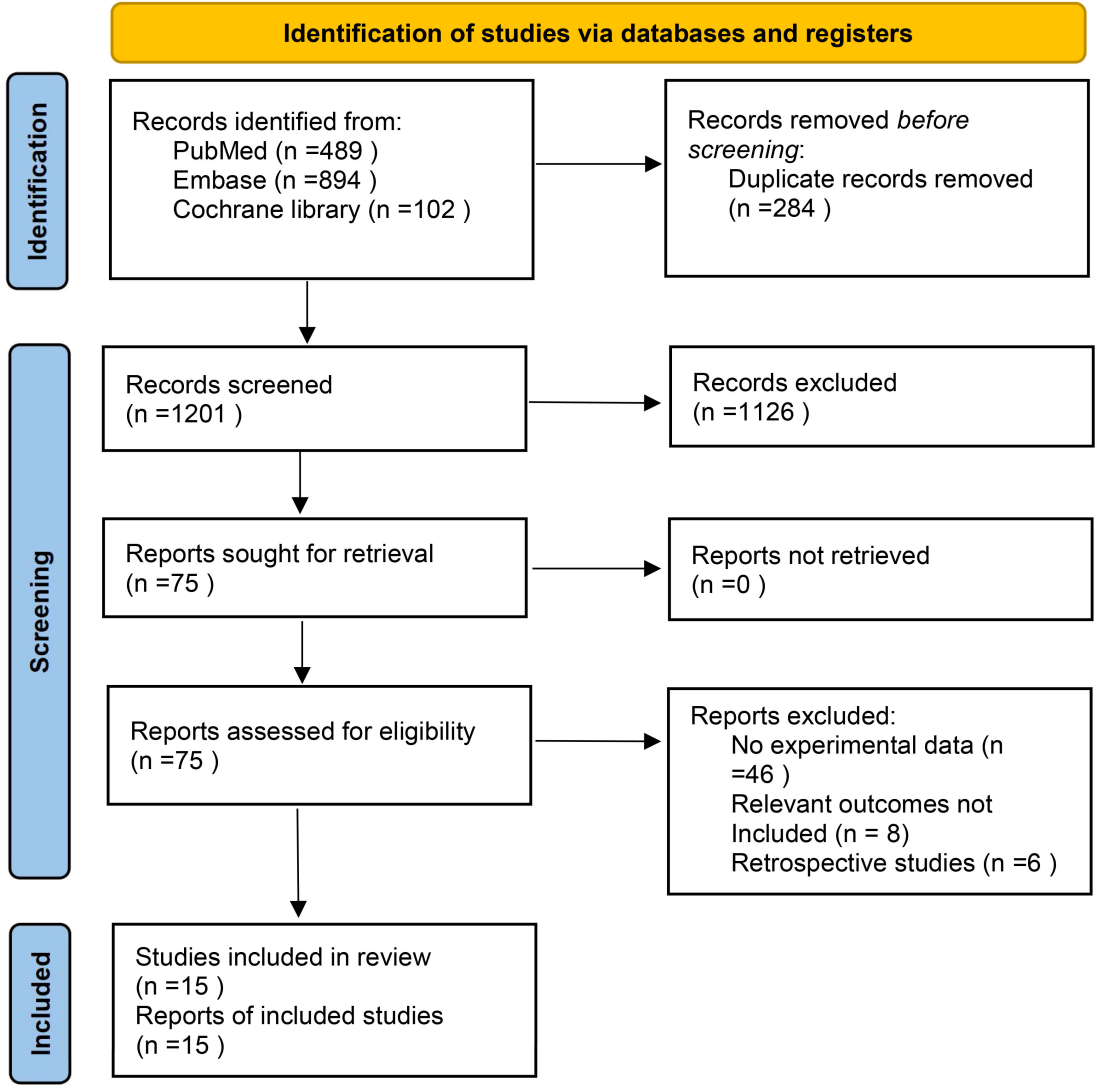
Flowchart of study selection.

In the selected database, a total of 1485 record**s** were retrieved and screened according to inclusion criteria and exclusion criteria, 284 duplicates were removed, and 1126 record**s** including case reports, animal experiments, reviews, systematic reviews and meta-analyses were excluded by reading titles and abstracts. Finally, 15 articles were included after reading the full-text articles (16–30). The literature screening process is shown in **Figure 1**. The characteristics and basic information of the included studies are shown in **Tables 2**, **3**.

**Table 2:** Characteristics of neoadjuvant systemic therapy studies in patients with hepatocellular carcinoma.

Study	Year	Trial Indentifier	Region	Sample Size	Study Phase	Intervention Model	Masking	Study Type	Randomization Method	Article Type	Neoadjuvant systemic therapy	pCR	MPR	Grade3-4 TRAEs	ORR	Surgical Rate	Surgical Delay
Shi, Y.H. et al. (16)	2021	NCT03867370	China	18	Ib/II	Single-arm	Open Label	Clinical Trial	NR	Conference abstract	Toripalimab± Lenvatinib	6.3%(1/16)	20%(3/15)	16.7%(3/18)	NR	100%(16/16)	0%(0/16)
Ho, W.J. et al. (17)	2021	NCT03299946	USA	15	Ib	Single-arm	Open Label	Clinical Trial	NR	Full text	Cabozantinib+ Nivolumab	8.3%(1/12)	42%(5/12)	13.3%(2/15)	NR	80%(12/15)	0%(0/14)
Chen, S. et al. (18)	2022	NCT04615143	China	11	II	Single-arm	Open Label	Clinical Trial	Non-Randomized	Conference abstract	Tislelizumab	9.1%(1/11)	18.2%(2/11)	0%(0/11)	18.2%(2/11)	100%(11/11)	0%(0/11)
D’Alessio, A. et al. (19)	2022	NCT03682276	UK	17	Ib	Single-arm	Open Label	Clinical Trial	NR	Conference abstract	ipilimumab+ Nivolumab	22%(2/9)	NR	7%(1/15)	23%(3/13)	NR	NR
Marron, T.U. et al. (20)	2022	NCT03916627	USA	21	II	Single-arm	Open Label	Clinical Trial	NR	Full text	Cemiplimab	15%(3/20)	20%(4/20)	10%(2/21)	15%(3/20)	95.2%(20/21)	4.8%(1/21)
Kaseb, A.O. et al. (21)	2022	NCT03222076	USA	30	II	Dual-arm	Open Label	Clinical Trial	Randomized	Full text	Nivolumab	22%(2/9)	33%(3/9)	23%(3/13)	23%(3/13)	100%(9/9)	0%(0/9)
											Nivolumab +Ipilimumab	27%(3/11)	27%(3/11)	43%(6/14)	0%(0/14)	100%(11/11)	0%(0/11)
Xia, Y. et al. (22)	2022	NCT04297202	China	18	II	Single-arm	Open Label	Clinical Trial	NR	Full text	Camrelizumab+ Apatinib	5.9%(1/17)	17.6%(3/17)	16.7%(3/18)	16.7%(3/18)	94.4%(17/18)	NR
Bai, X. et al. (23)	2022	NCT04930315	China	24	II	Single-arm	Open Label	Clinical Trial	Randomized	Conference abstract	Camrelizumab+ Apatinib	NR	33.3%(2/6)	NR	NR	75%(6/8)	0%(0/8)
Su, Y. et al. (24)	2023	NCT03510871	China	43		Single-arm	Open Label	Clinical Trial	NR	Conference abstract	Nivolumab+ ipilimumab	NR	33%(8/24)	44.2%(19/43)	NR	NR	NR
Sun, H.C. et al. (25)	2023	NCT04843943	China	30	II	Single-arm	Open Label	Clinical Trial	NR	Conference abstract	Sintilimab+ Bevacizumab	11.8%(2/17)	NR	23.3%(7/30)	26.7%(8/30)	56.7%(17/30)	NR
Song, T.Q (26).	2023	NCT04834986	China	24	II	Single-arm	Open Label	Clinical Trial	NR	Conference abstract	Tislelizumab+ lenvatinib	17.6%(3/17)	35.3%(6/17)	0%(0/24)	54.2%(13/24)	70.8%(17/24)	NR
Cheung, T. T. et al. (27)	2023	NCT05471674	China	20	II	Single-arm	Open Label	Clinical Trial	NR	Full text	Nivolumab	15.8%(3/19)	36.8%(7/19)	0%(0/20)	NR	95%(19/20)	NR
Huang, Z. et al. (28)	2023	NCT04888546	China	20	Ib	Single-arm	Open Label	Clinical Trial	NR	Conference abstract	TQB2450+ Anlotinib	23.1%(3/13)	NR	10%(2/20)	29.4%(5/17)	76.5%(13/17)	NR
T. Song (29)	2024	NCT05807776	China	23	II	Single-arm	Open Label	Clinical Trial	NR	Conference abstract	Tislelizumab± lenvatinib	30%(3/10)	20%(2/10)	6.3%(1/16)	25%(4/16)	62.5%(10/16)	NR
Ming, K. et al. (30)	2024	NCT04615143	China	14	II	Single-arm	Open Label	Clinical Trial	Non-Randomized	Conference abstract	Tislelizumab+ lenvatinib	25%(3/12)	NR	0%(0/14)	NR	85.7%(12/14)	NR

pCR, pathological complete response; MPR, major pathological response; Grade3–4 TRAEs, Grade 3–4 Treatment-Related Adverse Event Rate; ORR, objective response rate; NR, not reported.

**Table 3:** Basic information of the included studies.

Study	Gender (M/F)	Median Age (IQR)(years)	Underlying Liver Disease	Neoadjuvant Therapy Duration	Time to Surgery	Single/Multi-Center	Loss to Follow-Up/Discontinuation	Treatment Regimens	R0 Resection Rate	Progression After Surgery	Median Follow-Up	Adjuvant Therapy	Assessment Criteria
Shi, Y.H. et al. (16)	NR	NR	BCLC stage A HCC	3 weeks	Surgery at 21–28 days post-treatment	single-center	1 discontinuation (hyperglycemia)	ICI ± targeted drugs	NR	NR	NR	Toripalimab (48 weeks post-surgery)	RECIST 1.1
Ho, W.J. et al. (17)	13/2	NR	Mixed (viral hepatitis, NASH, alcohol-related)	8 weeks	≥28 days post-treatment	Multi-center	2 temporary holds (immune-related toxicity)	ICIs+targeted drugs	100% (12/12 margin-negative)	41.7%(5/12)	~1 year	None	RECIST 1.1
Chen, S. et al. (18)	NR	NR	NR	6 weeks	NR	single-center	All patients underwent surgery and follow-up	ICI monotherapy	NR	18.2%(2/11)	NR	Tislelizumab (1 year post-surgery)	RECIST 1.1
D’Alessio, A. et al. (19)	NR	NR	Child-Pugh A (some with HBV/HCV)	6 weeks	Post-treatment assessment at Day 43 (± 3 days)	Multi-center	No loss to follow-up/discontinuation	Dual ICIs	100% (12/12 margin-negative)	NR(ongoing trial)	Planned 2-year follow-up	Nivolumab (8 cycles post-surgery)	RECIST 1.1
Marron, T.U. et al. (20)	18/3	68	HBV 38%, HCV 23%, NASH 23%, alcohol-related 5%	6 weeks	Median 29 days (IQR 27–35) after treatment completion	single-center	1 delayed surgery (pneumonitis)	ICI monotherapy	100% (20/20 margin-negative)	25%(5/20)	~1 year	None	RECIST 1.1
Kaseb, A.O. et al. (21)	19/11	64 (53–69)	HBV 31% + HCV 23%, Child-Pugh A	6 weeks	Surgery after 6 weeks of enrollment	single-center	1 patient lost to follow-up prior to death 7/27 patients had surgical cancellations (non-AE reasons)	ICI monotherapy;Dual ICIs	100% (20/20)	Recurrence: 7/14 non-MPR patients No recurrence in MPR patients	24.6 months	Nivolumab (up to 2 years) or Nivolumab + Ipilimumab (up to 4 doses)	RECIST 1.1
Xia, Y. et al. (22)	17/1	54 (34–76)	HBV 83.3%	9 weeks	Surgery 46 days after neoadjuvant therapy initiation	single-center	2 patients withdrew consent 1 patient excluded due to disease progression 3 protocol violations	ICIs+targeted drugs	100% (17/17)	Recurrence: 7/14 non-MPR patients;1-year RFS:N16 53.85%	26.8 months	Camrelizumab + Apatinib (8 cycles)	RECIST 1.1 + mRECIST
Bai, X. et al. (23)	NR	NR	Resectable HCC (CNLC IIb/IIIa stage)	8 weeks	Surgery after completion of neoadjuvant therapy	Multi-center	No explicit loss to follow-up/discontinuation	ICIs+targeted drugs	100% (8/8)	Data immature	NR	Camrelizumab + Apatinib (8 cycles in perioperative group)	mRECIST
Su, Y. et al. (24)	37/6	60	HBsAg+: 60.5% Anti-HCV+: 11.6%	6–12 weeks	Surgical feasibility assessed after 6–12 weeks of enrollment	Multi-center	3 patients had early progression after 1 cycle No explicit loss to follow-up	Dual ICIs	NR	Estimated 2-year PFS: 47.1% (95% CI: 32.7–56.4)	NR	NR	RECIST 1.1
Sun, H.C. et al. (25)	NR	NR	BCLC stage B	NR	Median time from therapy initiation to surgery: 4.3 months (IQR: 2.3–5.6)	single-center	3 deaths reported; survival data immature No explicit loss to follow-up	ICIs+targeted drugs	100% (17/17)	6-month EFS: 76% 12-month EFS: 60%	15.3 months	Sintilimab + Bevacizumab (up to 12 months)	RECIST 1.1
Song, T.Q (26).	23/1	57	91.7% HBV+	12 weeks	Within 4 weeks post-treatment	single-center	3/24 patients refused surgery, 3 had disease progression, and 1 awaited surgery. No loss to follow-up.	ICIs+targeted drugs	70.8% (17/24)	NR	NR	Tislelizumab + lenvatinib for ≥6 months	RECIST 1.1 + mRECIST
Cheung, T. T. et al. (27)	16/4	65 (44–79)	70% HBV+	6 weeks	~6 weeks after last treatment	single-center	No explicit loss reported. All 20 patients completed neoadjuvant therapy.	ICI monotherapy	100% (19/19)	52.6% recurrence (10/19)	~15 months	NR	NR
Huang, Z. et al. (28)	19/1	61 (31–68)	HBV 80%	~9 weeks	Surgery performed within 4 weeks after treatment completion	single-center	No explicit loss reported. 17/20 patients were evaluable; 13 completed preoperative therapy and surgery.	ICIs+targeted drugs	100% (13/13)	NR	NR	Continued TQB2450 + anlotinib for 24 weeks	mRECIST
T. Song (29)	NR	NR	NR	6–12 weeks	NR	single-center	No explicit loss reported. 23 enrolled; 10 underwent surgery. 1 patient with grade 3 dermatomyositis resumed surgery after treatment.	ICI ± targeted drugs	NR	NR	NR	Tislelizumab ± lenvatinib post-surgery	RECIST 1.1 + mRECIST
Ming, K. et al. (30)	NR	NR	NR	6 weeks	6 weeks after enrollment	single-center	No loss reported. 14 enrolled; 12 underwent R0 resection, 2 awaited surgery.	ICIs+targeted drugs	85.7% (12/14)	NR	NR	Tislelizumab + lenvatinib for ≥1 year	RECIST 1.1

Gender (M/F), patients number(male/female); IQR, interquartile range; Child-pugh (A/B), liver function (grade A/grade B); NR, not reported.

### 3.2 Efficacy of neoadjuvant systemic therapy

**Figure 2:**
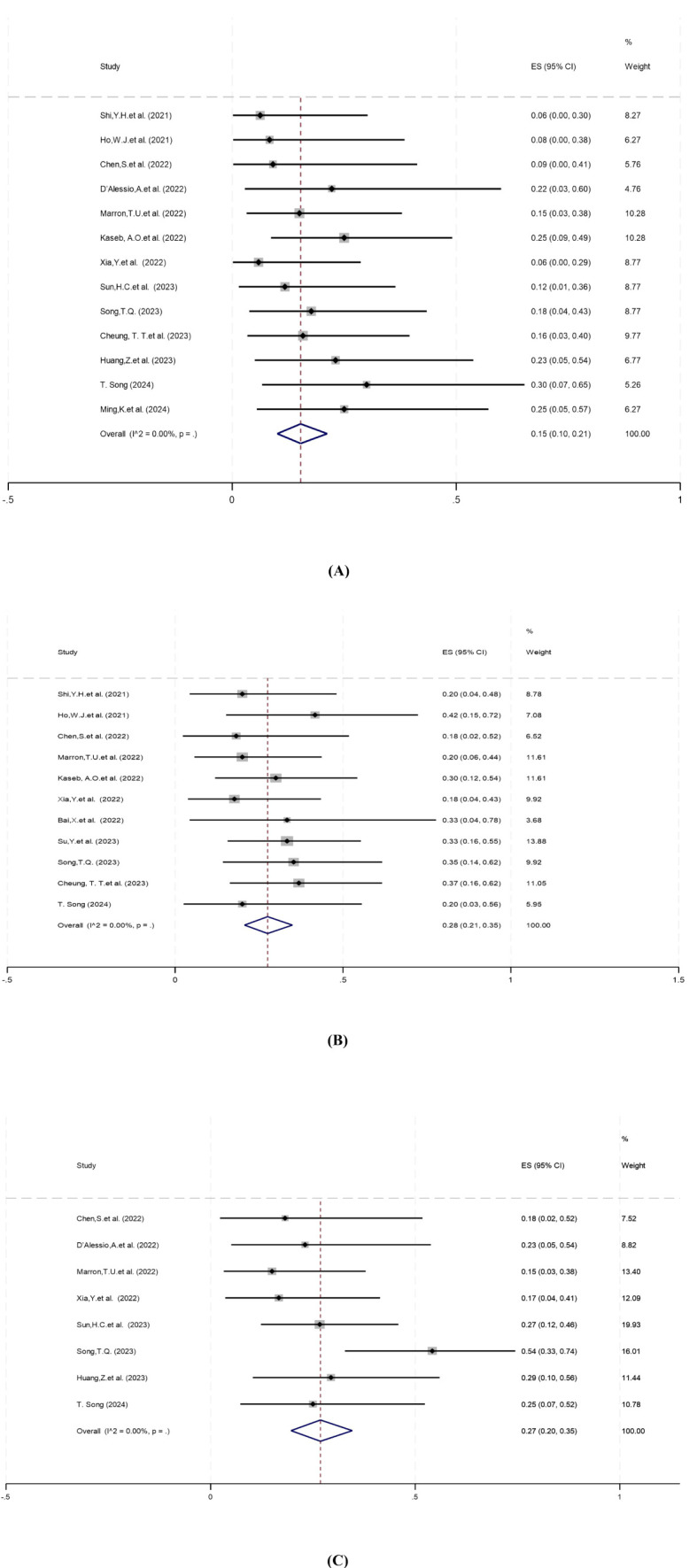
Forest plot of the efficacy of neoadjuvant systemic therapy in resectable hepatocellular carcinoma. **(A)** pCR, **(B)** MPR, **(C)** ORR. pCR, pathological complete response; MPR,major pathological response; ORR,objective response rate. ES, effect size (Proportion).

Pathologic complete response (pCR) was defined as the absence of viable tumor cells in the resected specimen, assessed by histopathological examination. Thirteen studies reported pCR rates ranging from 5.9% to 30%, with no statistical heterogeneity among the studies (*P*=0.86, *I^2^ =* 0.00%), so a meta-analysis using a fixed-effect model showed a pooled pCR rate of 15% (95%CI: 10%–21%) (**Figure 2A**).

Major pathologic response (MPR) was defined as ≤10% residual viable tumor in the resected specimen. Eleven studies reported MPR rates ranging from 18% to 42%. There was no statistical heterogeneity among the studies (*P*=0.89, *I^2^ =* 0.00%), and a fixed-effect model was used for meta-analysis. The results showed that the pooled MPR of neoadjuvant systemic therapy for resectable hepatocellular carcinoma was 28% (95% CI: 21%–35%) (**Figure 2B**).

Objective response rate (ORR) was defined as the proportion of patients achieving complete or partial response per RECIST 1.1 or mRECIST criteria (31, 32). Eight studies reported ORR ranging from 15% to 54%. There was no significant heterogeneity among the studies (*P*=0.19, *I^2^ =* 30.37%), so a fixed-effect model was used for meta-analysis. Results showed that the pooled ORR of neoadjuvant systemic therapy in resectable hepatocellular carcinoma was 27% (95% CI: 20%–35%) (**Figure 2C**).

### 3.3 Safety of neoadjuvant systemic therapy

**Figure 3:**
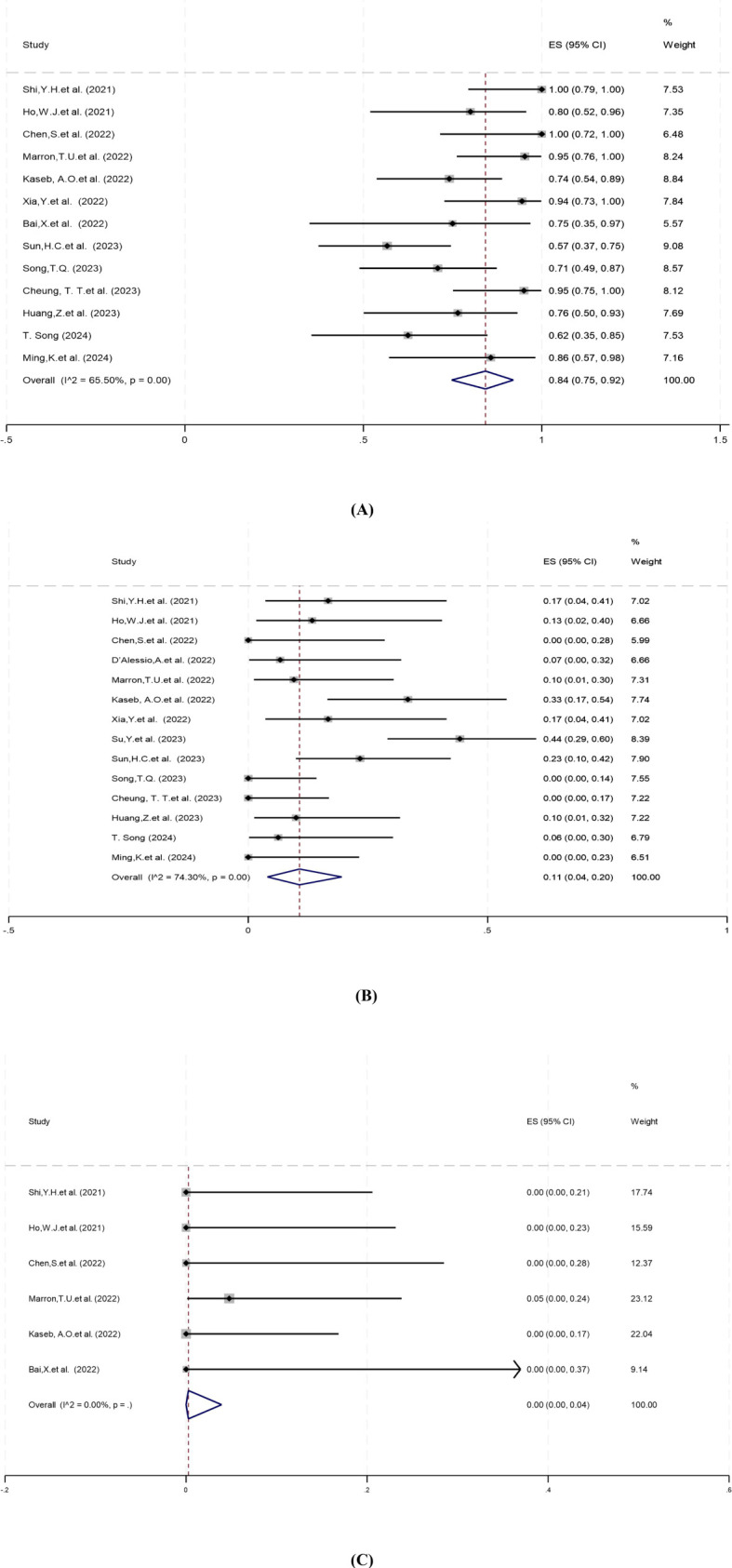
Forest plot of the safety of neoadjuvant systemic therapy in resectable hepatocellular carcinoma. **(A)** Surgical resection rate, **(B)** Grade 3–4 TRAEs, **(C)** Surgery delay rates. TRAEs, treatment-related adverse events. ES, effect size (Proportion).

Thirteen studies reported surgical resection rates ranging from 57% to 100%. There was statistical heterogeneity among the studies (*P*=0.00, *I^2^ =* 65.50%), hence a random-effects model was used for meta-analysis. The pooled results showed that the surgical resection rate of neoadjuvant systemic therapy was 84% (95% CI: 75%–92%) (**Figure 3A**).

Treatment-related adverse events (TRAEs), as defined by the National Cancer Institute’s Common Terminology Criteria for Adverse Events (CTCAE) version 5.0, is associated with the safety of neoadjuvant systemic therapy (33). Fourteen studies reported incidence of grade 3–4 TRAEs ranging from 0% to 44%. There was significant heterogeneity among studies (*P*=0.00, *I^2^ =* 74.30%), and a random-effects model was employed for meta-analysis. The results showed that the pooled incidence of grade 3–4 TRAEs of neoadjuvant systemic therapy for resectable hepatocellular carcinoma was 11% (95% CI: 4%–20%) (**Figure 3B**).

Surgical delay rate was defined as the ratio of patients whose surgery was delayed due to adverse events caused by neoadjuvant systemic therapy to all patients expected to have surgery. Six studies reported surgery delay rates ranging from 0% to 5%. There was no significant heterogeneity among the studies (*P*=0.94, *I^2^ =* 0.00%), and a fixed-effect model was applied for meta-analysis. The pooled results showed that the surgical delay rate of neoadjuvant systemic therapy was 0.00% (95% CI: 0.00%–4%) (**Figure 3C**).

### 3.4 Subgroup analysis

Currently, the neoadjuvant systemic therapy for HCC mainly includes three regimens: ICI monotherapy, dual ICI combination, and immunotherapy combined with targeted therapy. Subgroup analysis was performed to determine the possible sources of heterogeneity, and to find out differences in efficacy and safety among different treatment regimens, and whether they had different effects on clinical outcomes (pCR, MPR, Grade 3–4 TRAEs, ORR, surgical resection rate, surgical delay rate).

**Figure 4:**
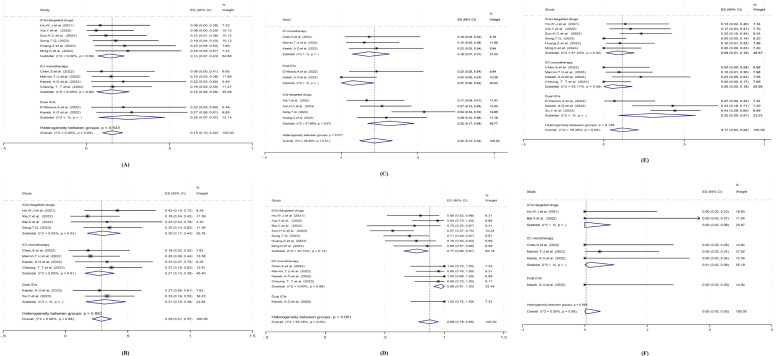
Subgroup analysis based on different neoadjuvant systemic therapy regimens for **(A)** pCR, **(B)** MPR, **(C)** ORR, **(D)** Surgical resection rate, **(E)** Grade 3–4 TRAEs, **(F)** Surgery delay rates. pCR, pathological complete response; MPR, major pathological response; ORR, objective response rate; TRAEs, treatment-related adverse events. ES, effect size (Proportion). Note: Heterogeneity values are not displayed if there are three or fewer studies in the subgroup.

For different treatment regimens, the pCR rate for dual ICI therapy was greater than that for ICI monotherapy, which was greater than immunotherapy combined with targeted therapy, but no statistically significant difference was observed among the groups(Q=1.22, df=2, *P*=0.543) (**Figure 4A**). The MPR rate for dual ICI therapy was greater than that for immunotherapy combined with targeted therapy, which was greater than that for ICI monotherapy, but no statistically significant difference was observed among the groups(Q=0.25, df=2, *P*=0.883) (**Figure 4B**). The ORR for immunotherapy combined with targeted therapy was greater than that for ICI monotherapy, which was greater than that for dual ICI therapy, and showed statistically significant differences among the groups(Q=5.29, df=2, *P*=0.071) (**Figure 4C**). The surgical resection rate was greater for dual ICI therapy than for ICI monotherapy, which was greater than that for immunotherapy combined with targeted therapy, and showed statistically significant differences among the groups(Q=14.05, df=2, *P*=0.001) (**Figure 4D**). The incidence of grade 3–4 TRAEs was greater for dual ICI therapy than for immunotherapy combined with targeted therapy, which was greater than that for ICI monotherapy, but no statistically significant difference was observed among the groups(Q=4.12, df=2, *P*=0.128) (**Figure 4E**). The surgical delay rate was higher for ICI monotherapy than for immunotherapy combined with targeted therapy and dual ICI therapy, but no statistically significant differences were observed among the groups(Q=0.29, df=2, *P*=0.864) (**Figure 4F**).

### 3.5 Publication bias

**Figure 5:**
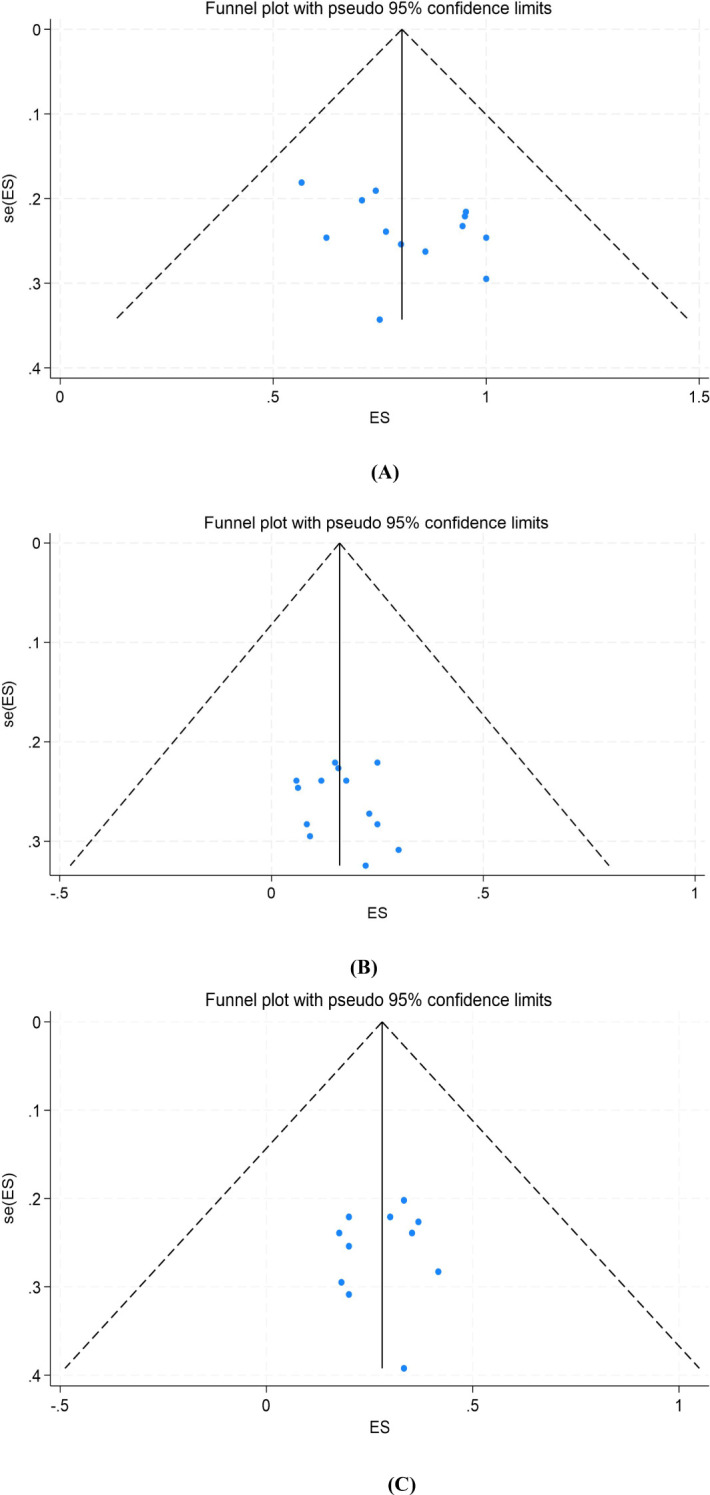
Funnel plot of the meta-analysis. **(A)** Surgical resection rate, **(B)** pCR, **(C)** MPR. pCR, pathological complete response; MPR, major pathological response. ES, effect size (Proportion). se, standard error.

To assess potential publication bias, we constructed funnel plots and performed Egger’s test for surgical resection rate, pCR, and MPR. The funnel plots showed symmetrical distribution, and the corresponding *P* values of Egger’s test were 0.157, 0.456 and 0.768, respectively, suggesting that there was no publication bias (**Figure 5**).

## 4 Discussion

The results of this meta-analysis preliminarily suggest that neoadjuvant systemic therapy may have an advantage in resectable HCC. First, regarding its efficacy, the pooled results showed an ORR of 27% (95% CI: 20%-35%), with a maximum reported ORR of 54.2% (26). The pooled pCR and MPR results were 15% (95%CI: 10%-21%) and 28% (95%CI: 21%-35%), respectively, with the reported maximum pCR and MPR values of 30% and 42% (17, 29), respectively, a result consistent with neoadjuvant systemic therapy results for other tumor types (34–37). However, given that most clinical studies are ongoing, data on patient survival after tumor resection is limited. Only four articles provided statistical data, among which Kaseb, A.O. et al. (21) reported PFS (Progression-Free Survival) of 9.4 months (95%CI: 1.47-not estimable[NE]) for nivolumab and 19.53 months (95%CI: 2.33-NE) for nivolumab plus ipilimumab. Other studies (22, 25) reported EFS (Event-Free Survival) and RFS (Recurrence-Free Survival) data, with a median EFS of 13.8 months (95% CI: 10.3-17.3) and a 1-year RFS of 53.85% (95% CI: 24.77%-75.99%). However, due to insufficient follow-up time, no studies have reported OS (Overall Survival) data. In other tumors, a significant correlation between pathological response and survival has been demonstrated (38, 39). Statistical analysis was also performed in this study, as Ho, W.J et al. (17) found a correlation between achieving MPR and long-term DFS (Disease-Free Survival), with DFS intervals of more than 230 days in all patients to date. Kaseb, A. O. et al. (21) also reported a significant correlation between pathological response and RFS (*P*=0.049). Six patients with pathological response did not have recurrence after a median follow-up of 26.8 months, while seven of the other 14 patients without pathological response had recurrence. Xia, Y et al. (22) found that the RFS of patients with MPR/pCR was higher than that of patients non-MPR/pCR.

Postoperative recurrence of HCC is the main obstacle to the improvement of the efficacy of surgical resection, and it is also the common cause of disease progression (12, 40). Neoadjuvant systemic therapy can reduce tumor burden, eliminate intrahepatic micrometastases in advance, and improve the prognosis of patients (41, 42). PD-1/PD-L1 is involved in tumor immune escape. The activation of PD-1/PD-L1 signaling pathway can lead to the formation of immunosuppressive tumor microenvironment, so that tumor cells can escape the body’s immune surveillance and killing (43, 44). In the immune response of tumor cells, the induced apoptosis of tumor antigen-specific T lymphocytes is involved in tumor immune evasion (45). PD-1/PD-L1 plays an important role in inhibiting the activation and proliferation of T lymphocytes. After the anti-tumor immunity of T cells is activated by ICI antibodies, the immune memory effect of T cells is often long-lasting, which can continue to play an immune surveillance role after surgical resection, thereby helping to reduce tumor recurrence (46). Marron, T.U. et al. (20) found that patients with more than 50% tumor necrosis after cemiplimab treatment showed stronger immune infiltration than those with minimal necrosis in surgical samples. However, the incidence of disease progression in PD-1/PD-L1 antibody immune monotherapy is high, generally around 40% (47–50); therefore, immune monotherapy is not an ideal choice for neoadjuvant therapy. Xu J et al. (51) found that apatinib combined with camrelizumab (PD-1 antibody) had a good anti-tumor effect in phase II clinical study, with an ORR of 34.3% and a disease progression rate of 18.6%. Immunotherapy combined with targeted therapy, such as small molecule TKI or bevacizumab, has shown stronger anti-tumor effect and significantly reduced the incidence of disease progression, which is expected to become a more reliable choice of neoadjuvant systemic therapy. In a randomized controlled clinical trial reported by Kaseb, A.O. et al. (21), 30 patients were screened, of whom 27 patients were randomly divided into ICI monotherapy group (receiving nivolumab monotherapy) (n=13) and dual ICI group (receiving nivolumab plus ipilimumab) (n=14). Among the 25 patients who completed the treatment, the median PFS was 9.4 months in the former group and 19.53 months in the latter group. It is suggested that dual ICI therapy may have better long-term survival benefits.

Regarding the safety of neoadjuvant systemic therapy in resectable HCC, the pooled results for grade 3–4 TRAEs were 11% (95% CI: 4% to 20%), with the highest incidence of 44.2% reported by Su, Y. et al. (24). Common adverse events included pneumonia, drug-induced hepatitis, pruritus, maculopapular rash, severe muscle weakness, elevated lipase and leukopenia. Most of the adverse events were manageable, some of which could be resolved by drug withdrawal, and others could be relieved by steroid therapy. Overall, neoadjuvant systemic therapy for HCC has shown a safety profile similar to previous studies in gastrointestinal tumors (52). In addition, the only randomized controlled trial reported by Kaseb, A.O, et al. (21) in this study found a higher incidence of grade 3–4 TRAEs with nivolumab plus ipilimumab than with nivolumab alone (23% vs 43%), indicating that different combinations may lead to different outcomes. Different combination regimens should be optimized to maximize patient benefit. In addition, the pooled surgical resection rate after neoadjuvant systemic therapy was 84% (95% CI: 75%-92%). Although the majority of patients underwent surgery as scheduled, a small number of patients may not tolerate surgery due to toxicity caused by neoadjuvant therapy or lose the opportunity for surgery due to tumor progression. Therefore, neoadjuvant systemic therapy for patients with resectable HCC requires MDT (Multi-Disciplinary Treatment) to develop an individualized and comprehensive treatment plan to maximize patient benefits, ensure treatment safety, and improve treatment outcomes (53).

This study found that there was high heterogeneity in surgical resection rate (I²=65.5%) and grade 3–4 TRAEs (I²=74.3%), which may be related to the study type (most of the included studies were single-arm studies). At the same time, we also conducted a subgroup analysis (according to the three different treatment regimens of ICI monotherapy, dual ICIs and targeted–immunotherapy combination) and found that the reason for high heterogeneity may be related to different treatment regimens. It may also be related to the tumor stage, liver function, surgical standard difference, physical condition and drug dose of patients. The results of subgroup analysis showed that for different neoadjuvant systemic treatment regimens, the surgical resection rate of dual ICIs was better than that of ICI monotherapy, and ICI monotherapy was better than that of targeted–immunotherapy combination. In terms of ORR, targeted–immunotherapy combination was superior to ICI monotherapy, and ICI monotherapy was superior to dual ICIs. Certainly, in terms of the incidence of grade 3–4 TRAEs, the dual ICI combination therapy was the highest and the ICI monotherapy was the lowest. Immunotherapy and targeted therapy have been widely used in the clinical application of HCC (54, 55). Single therapy has limited clinical effect and may also produce drug resistance. Several studies have shown that combination therapy can lead to better treatment outcomes. For example, the IMbrave150 trial (56) showed significant improvement in clinical outcomes with a 1-year survival rate of 67.2% and mPFS of 6.8 months in previously untreated patients with advanced HCC treated with atezolizumab plus bevacizumab. It is currently approved for first-line treatment of advanced HCC. Other combinations such as sintilimab plus bevacizumab and camrelizumab plus apatinib, have also shown promising results (51, 57). In addition, the IMbrave050 (58) phase III clinical study results are expected to show that the combination of ICIs plus antiangiogenic drugs (T+A regimen) can significantly reduce the risk of recurrence, distant metastasis, or death by 28% in patients with HCC after radical treatment (including surgical resection or ablation), showing a clear survival benefit. Mechanistically, immunomodulatory drugs have been found to restore the immune-supporting microenvironment, whereas anti-VEGF agents such as bevacizumab improve immunosuppression and help restore vascular normalization for effective administration, allowing lower doses of ICIs to reduce adverse effects (59). In our subgroup analysis, the ORR of the targeted–immunotherapy combination was significantly superior, but the surgical resection rate was lower than in other subgroups. The long-term survival benefit after surgery still needs to be assessed, which will provide effective clinical evidence for choosing the best treatment strategy in the future.

This meta-analysis has certain limitations. On the one hand, the included studies differed in study design, treatment regimen, and patient inclusion criteria, etc. (including lack of data on tumor size and number at study enrollment and/or at time of post-neoadjuvant evaluation for surgery), resulting in heterogeneity. On the other hand, most of the data came from conference abstracts, no unified standard for R0 resection rate has been reported(as shown in **Table 3**), and there was also a lack of long-term follow-up survival indicators such as OS, DFS, RFS, and PFS, which may affect the reliability of the results. Because of the lack of these data, we could not perform a comprehensive analysis, and therefore, whether neoadjuvant systemic therapy contributes to the long-term benefit of HCC patients needs to be further confirmed.

## 5 Conclusion

In conclusion, our meta-analysis demonstrated the efficacy and safety of neoadjuvant systemic therapy in patients with resectable HCC, providing evidence for its future clinical application. However, due to limited clinical data, large-scale and multicenter randomized controlled trials are needed to confirm this conclusion in the future.

## Data Availability

The datasets presented in this study can be found in online repositories. The names of the repository/repositories and accession number(s) can be found in the article/**Supplementary Material**.
