# Supplementary material for: Efficacy and safety of neoadjuvant systemic therapy in resectable hepatocellular carcinoma: a Systematic Review and meta-analysis
Source: Front Oncol. 2025 May 9;15:1504917. doi: 10.3389/fonc.2025.1504917 (PMC12098073; doi:10.3389/fonc.2025.1504917)
Supplement: Supplementary file 1 [file Table1.docx]

**Efficacy and safety of neoadjuvant systemic therapy in resectable hepatocellular carcinoma: a systematic review and meta-analysis**

**Dongdong Wu, Ning Liu, Hao Dong, Kan Zhou, Lei Du, Ying Li, Yanjun Chao and Fuping Ma**

**Search strategy in PubMed**

(("Carcinoma, Hepatocellular"[Mesh]) OR (((((((((((((((((((Carcinomas, Hepatocellular[Title/Abstract]) OR (Hepatocellular Carcinomas[Title/Abstract])) OR (Liver Cell Carcinoma, Adult[Title/Abstract])) OR (Liver Cancer, Adult[Title/Abstract])) OR (Adult Liver Cancer[Title/Abstract])) OR (Adult Liver Cancers[Title/Abstract])) OR (Cancer, Adult Liver[Title/Abstract])) OR (Cancers, Adult Liver[Title/Abstract])) OR (Liver Cancers, Adult[Title/Abstract])) OR (Liver Cell Carcinoma[Title/Abstract])) OR (Carcinoma, Liver Cell[Title/Abstract])) OR (Carcinomas, Liver Cell[Title/Abstract])) OR (Cell Carcinoma, Liver[Title/Abstract])) OR (Cell Carcinomas, Liver[Title/Abstract])) OR (Liver Cell Carcinomas[Title/Abstract])) OR (Hepatocellular Carcinoma[Title/Abstract])) OR (Hepatoma[Title/Abstract])) OR (Hepatomas[Title/Abstract])) OR (HCC[Title/Abstract]))) AND (("Neoadjuvant Therapy"[Mesh]) OR ((((((((((((((((((((((((((((((((((((((((((((((((Neoadjuvant Therapies[Title/Abstract]) OR (Therapy, Neoadjuvant[Title/Abstract])) OR (Neoadjuvant Treatment[Title/Abstract])) OR (Neoadjuvant Treatments[Title/Abstract])) OR (Treatment, Neoadjuvant[Title/Abstract])) OR (Neoadjuvant Chemoradiotherapy[Title/Abstract])) OR (Chemoradiotherapy, Neoadjuvant[Title/Abstract])) OR (Neoadjuvant Chemoradiotherapies[Title/Abstract])) OR (Neoadjuvant Chemoradiation Therapy[Title/Abstract])) OR (Chemoradiation Therapy, Neoadjuvant[Title/Abstract])) OR (Neoadjuvant Chemoradiation Therapies[Title/Abstract])) OR (Therapy, Neoadjuvant Chemoradiation[Title/Abstract])) OR (Neoadjuvant Chemoradiation Treatment[Title/Abstract])) OR (Chemoradiation Treatment, Neoadjuvant[Title/Abstract])) OR (Neoadjuvant Chemoradiation Treatments[Title/Abstract])) OR (Treatment, Neoadjuvant Chemoradiation[Title/Abstract])) OR (Neoadjuvant Chemoradiation[Title/Abstract])) OR (Chemoradiation, Neoadjuvant[Title/Abstract])) OR (Neoadjuvant Chemoradiations[Title/Abstract])) OR (Neoadjuvant Radiotherapy[Title/Abstract])) OR (Neoadjuvant Radiotherapies[Title/Abstract])) OR (Radiotherapy, Neoadjuvant[Title/Abstract])) OR (Neoadjuvant Radiation Treatment[Title/Abstract])) OR (Neoadjuvant Radiation Treatments[Title/Abstract])) OR (Radiation Treatment, Neoadjuvant[Title/Abstract])) OR (Treatment, Neoadjuvant Radiation[Title/Abstract])) OR (Neoadjuvant Radiation Therapy[Title/Abstract])) OR (Neoadjuvant Radiation Therapies[Title/Abstract])) OR (Radiation Therapy, Neoadjuvant[Title/Abstract])) OR (Therapy, Neoadjuvant Radiation[Title/Abstract])) OR (Neoadjuvant Radiation[Title/Abstract])) OR (Neoadjuvant Radiations[Title/Abstract])) OR (Radiation, Neoadjuvant[Title/Abstract])) OR (Neoadjuvant Chemotherapy[Title/Abstract])) OR (Chemotherapy, Neoadjuvant[Title/Abstract])) OR (Neoadjuvant Chemotherapies[Title/Abstract])) OR (Neoadjuvant Chemotherapy Treatment[Title/Abstract])) OR (Chemotherapy Treatment, Neoadjuvant[Title/Abstract])) OR (Neoadjuvant Chemotherapy Treatments[Title/Abstract])) OR (Treatment, Neoadjuvant Chemotherapy[Title/Abstract])) OR (Neoadjuvant Systemic Therapy[Title/Abstract])) OR (Neoadjuvant Systemic Therapies[Title/Abstract])) OR (Systemic Therapy, Neoadjuvant[Title/Abstract])) OR (Therapy, Neoadjuvant Systemic[Title/Abstract])) OR (Neoadjuvant Systemic Treatment[Title/Abstract])) OR (Neoadjuvant Systemic Treatments[Title/Abstract])) OR (Systemic Treatment, Neoadjuvant[Title/Abstract])) OR (Treatment, Neoadjuvant Systemic[Title/Abstract])))

**Search strategy in Cochrane library**

#1 MeSH descriptor: [Carcinoma, Hepatocellular] explode all trees

#2 (Carcinomas, Hepatocellular):ti,ab,kw OR (Hepatocellular Carcinomas):ti,ab,kw OR (Liver Cell Carcinoma, Adult):ti,ab,kw OR (Liver Cancer, Adult):ti,ab,kw OR (Adult Liver Cancer):ti,ab,kw OR (Adult Liver Cancers):ti,ab,kw OR (Cancer, Adult Liver):ti,ab,kw OR (Cancers, Adult Liver):ti,ab,kw OR (Liver Cancers, Adult):ti,ab,kw OR (Liver Cell Carcinoma):ti,ab,kw OR (Carcinoma, Liver Cell):ti,ab,kw OR (Carcinomas, Liver Cell):ti,ab,kw OR (Cell Carcinoma, Liver):ti,ab,kw OR (Cell Carcinomas, Liver):ti,ab,kw OR (Liver Cell Carcinomas):ti,ab,kw OR (Hepatocellular Carcinoma):ti,ab,kw OR (Hepatoma):ti,ab,kw OR (Hepatomas):ti,ab,kw OR (HCC):ti,ab,kw

#3 #1 OR #2

#4 Resectable:ti,ab,kw

#5 #3 AND #4

#6 MeSH descriptor: [Neoadjuvant Therapy] explode all trees

#7 (Neoadjuvant Therapies):ti,ab,kw OR (Therapy, Neoadjuvant):ti,ab,kw OR (Neoadjuvant Treatment):ti,ab,kw OR (Neoadjuvant Treatments):ti,ab,kw OR (Treatment, Neoadjuvant):ti,ab,kw OR (Neoadjuvant Chemoradiotherapy):ti,ab,kw OR (Chemoradiotherapy, Neoadjuvant):ti,ab,kw OR (Neoadjuvant Chemoradiotherapies):ti,ab,kw OR (Neoadjuvant Chemoradiation Therapy):ti,ab,kw OR (Chemoradiation Therapy, Neoadjuvant):ti,ab,kw OR (Neoadjuvant Chemoradiation Therapies):ti,ab,kw OR (Therapy, Neoadjuvant Chemoradiation):ti,ab,kw OR (Neoadjuvant Chemoradiation Treatment):ti,ab,kw OR (Chemoradiation Treatment, Neoadjuvant):ti,ab,kw OR (Neoadjuvant Chemoradiation Treatments):ti,ab,kw OR (Treatment, Neoadjuvant Chemoradiation):ti,ab,kw OR (Neoadjuvant Chemoradiation):ti,ab,kw OR (Chemoradiation, Neoadjuvant):ti,ab,kw OR (Neoadjuvant Chemoradiations):ti,ab,kw OR (Neoadjuvant Radiotherapy):ti,ab,kw OR (Neoadjuvant Radiotherapies):ti,ab,kw OR (Radiotherapy, Neoadjuvant):ti,ab,kw OR (Neoadjuvant Radiation Treatment):ti,ab,kw OR (Neoadjuvant Radiation Treatments):ti,ab,kw OR (Radiation Treatment, Neoadjuvant):ti,ab,kw OR (Treatment, Neoadjuvant Radiation):ti,ab,kw OR (Neoadjuvant Radiation Therapy):ti,ab,kw OR (Neoadjuvant Radiation Therapies):ti,ab,kw OR (Radiation Therapy, Neoadjuvant):ti,ab,kw OR (Therapy, Neoadjuvant Radiation):ti,ab,kw OR (Neoadjuvant Radiation):ti,ab,kw OR (Neoadjuvant Radiations):ti,ab,kw OR (Radiation, Neoadjuvant):ti,ab,kw OR (Neoadjuvant Chemotherapy):ti,ab,kw OR (Chemotherapy, Neoadjuvant):ti,ab,kw OR (Neoadjuvant Chemotherapies):ti,ab,kw OR (Neoadjuvant Chemotherapy Treatment):ti,ab,kw OR (Chemotherapy Treatment, Neoadjuvant):ti,ab,kw OR (Neoadjuvant Chemotherapy Treatments):ti,ab,kw OR (Treatment, Neoadjuvant Chemotherapy):ti,ab,kw OR (Neoadjuvant Systemic Therapy):ti,ab,kw OR (Neoadjuvant Systemic Therapies):ti,ab,kw OR (Systemic Therapy, Neoadjuvant):ti,ab,kw OR (Therapy, Neoadjuvant Systemic):ti,ab,kw OR (Neoadjuvant Systemic Treatment):ti,ab,kw OR (Neoadjuvant Systemic Treatments):ti,ab,kw OR (Systemic Treatment, Neoadjuvant):ti,ab,kw OR (Treatment, Neoadjuvant Systemic):ti,ab,kw

#8 #6 OR #7

#9 #5 AND #8

**Search strategy in Embase**

#1 'liver cell carcinoma'/exp

#2 'Carcinomas, Hepatocellular':ab,ti OR 'Hepatocellular Carcinomas':ab,ti OR 'Liver Cell Carcinoma, Adult':ab,ti OR 'Liver Cancer, Adult':ab,ti OR 'Adult Liver Cancer':ab,ti OR 'Adult Liver Cancers':ab,ti OR 'Cancer, Adult Liver':ab,ti OR 'Cancers, Adult Liver':ab,ti OR 'Liver Cancers, Adult':ab,ti OR 'Liver Cell Carcinoma':ab,ti OR 'Carcinoma, Liver Cell':ab,ti OR 'Carcinomas, Liver Cell':ab,ti OR 'Cell Carcinoma, Liver':ab,ti OR 'Cell Carcinomas, Liver':ab,ti OR 'Liver Cell Carcinomas':ab,ti OR 'Hepatocellular Carcinoma':ab,ti OR 'Hepatoma':ab,ti OR 'Hepatomas':ab,ti OR 'HCC':ab,ti OR 'Carcinoma, Hepatocellular':ab,ti

#3 #1 OR #2

#4 'neoadjuvant therapy'/exp

#5 'Neoadjuvant Therapies':ab,ti OR 'Therapy, Neoadjuvant':ab,ti OR 'Neoadjuvant Treatment':ab,ti OR 'Neoadjuvant Treatments':ab,ti OR 'Treatment, Neoadjuvant':ab,ti OR 'Neoadjuvant Chemoradiotherapy':ab,ti OR 'Chemoradiotherapy, Neoadjuvant':ab,ti OR 'Neoadjuvant Chemoradiotherapies':ab,ti OR 'Neoadjuvant Chemoradiation Therapy':ab,ti OR 'Chemoradiation Therapy, Neoadjuvant':ab,ti OR 'Neoadjuvant Chemoradiation Therapies':ab,ti OR 'Therapy, Neoadjuvant Chemoradiation':ab,ti OR 'Neoadjuvant Chemoradiation Treatment':ab,ti OR 'Chemoradiation Treatment, Neoadjuvant':ab,ti OR 'Neoadjuvant Chemoradiation Treatments':ab,ti OR 'Treatment, Neoadjuvant Chemoradiation':ab,ti OR 'Neoadjuvant Chemoradiation':ab,ti OR 'Chemoradiation, Neoadjuvant':ab,ti OR 'Neoadjuvant Chemoradiations':ab,ti OR 'Neoadjuvant Radiotherapy':ab,ti OR 'Neoadjuvant Radiotherapies':ab,ti OR 'Radiotherapy, Neoadjuvant':ab,ti OR 'Neoadjuvant Radiation Treatment':ab,ti OR 'Neoadjuvant Radiation Treatments':ab,ti OR 'Radiation Treatment, Neoadjuvant':ab,ti OR 'Treatment, Neoadjuvant Radiation':ab,ti OR 'Neoadjuvant Radiation Therapy':ab,ti OR 'Neoadjuvant Radiation Therapies':ab,ti OR 'Radiation Therapy, Neoadjuvant':ab,ti OR 'Therapy, Neoadjuvant Radiation':ab,ti OR 'Neoadjuvant Radiation':ab,ti OR 'Neoadjuvant Radiations':ab,ti OR 'Radiation, Neoadjuvant':ab,ti OR 'Neoadjuvant Chemotherapy':ab,ti OR 'Chemotherapy, Neoadjuvant':ab,ti OR 'Neoadjuvant Chemotherapies':ab,ti OR 'Neoadjuvant Chemotherapy Treatment':ab,ti OR 'Chemotherapy Treatment, Neoadjuvant':ab,ti OR 'Neoadjuvant Chemotherapy Treatments':ab,ti OR 'Treatment, Neoadjuvant Chemotherapy':ab,ti OR 'Neoadjuvant Systemic Therapy':ab,ti OR 'Neoadjuvant Systemic Therapies':ab,ti OR 'Systemic Therapy, Neoadjuvant':ab,ti OR 'Therapy, Neoadjuvant Systemic':ab,ti OR 'Neoadjuvant Systemic Treatment':ab,ti OR 'Neoadjuvant Systemic Treatments':ab,ti OR 'Systemic Treatment, Neoadjuvant':ab,ti OR 'Treatment, Neoadjuvant Systemic':ab,ti

#6 #4 OR #5

#7 #3 AND #6
